# The Spatiotemporal Coupling: Regional Energy Failure and Aberrant Proteins in Neurodegenerative Diseases

**DOI:** 10.3390/ijms222111304

**Published:** 2021-10-20

**Authors:** Assunta Virtuoso, Anna Maria Colangelo, Nicola Maggio, Uri Fennig, Nitai Weinberg, Michele Papa, Ciro De Luca

**Affiliations:** 1 Laboratory of Neuronal Networks, Department of Mental and Physical Health and Preventive Medicine, University of Campania ‘‘Luigi Vanvitelli”, 80138 Naples, Italy; assunta.virtuoso@unicampania.it (A.V.); ciro.deluca@unicampania.it (C.D.L.); 2 SYSBIO Centre of Systems Biology ISBE-IT, University of Milano-Bicocca, 20126 Milan, Italy; annamaria.colangelo@unimib.it; 3 Laboratory of Neuroscience “R. Levi-Montalcini”, Department of Biotechnology and Biosciences, University of Milano-Bicocca, 20126 Milan, Italy; 4 Department of Neurology and Neurosurgery, Sackler Faculty of Medicine, Sagol School of Neuroscience, Tel Aviv University, Tel Aviv 6997801, Israel; Nicola.maggio@sheba.health.gov.il (N.M.); uri.fennig@sheba.health.gov.il (U.F.); nitai.weinberg@sheba.health.gov.il (N.W.); 5 Department of Neurology, The Chaim Sheba Medical Center at Tel HaShomer, Ramat Gan 52662, Israel

**Keywords:** systems biology, neurodegenerative diseases, synaptic plasticity, metabolism, extra-cellular matrix, neurovascular unit, astrocytes, microglia, oligodendroglia, neurons

## Abstract

The spatial and temporal coordination of each element is a pivotal characteristic of systems, and the central nervous system (CNS) is not an exception. Glial elements and the vascular interface have been considered more recently, together with the extracellular matrix and the immune system. However, the knowledge of the single-element configuration is not sufficient to predict physiological or pathological long-lasting changes. Ionic currents, complex molecular cascades, genomic rearrangement, and the regional energy demand can be different even in neighboring cells of the same phenotype, and their differential expression could explain the region-specific progression of the most studied neurodegenerative diseases. We here reviewed the main nodes and edges of the system, which could be studied to develop a comprehensive knowledge of CNS plasticity from the neurovascular unit to the synaptic cleft. The future goal is to redefine the modeling of synaptic plasticity and achieve a better understanding of neurological diseases, pointing out cellular, subcellular, and molecular components that couple in specific neuroanatomical and functional regions.

## 1. Introduction

The brain is a system in which the constituents are connected such that a change in the state of one can have some effect on the states or connections of the other components, and these interactions generate unforeseeable emergent properties [1]. A common feature of the systems, the central nervous system (CNS) as well as the immune system (IS), is the crucial need for the spatial and temporal concurrency of events to trigger long-term changes. These, in most cases, are sustained by substantial ionic fluctuations, generating complex molecular cascade and involving protein de novo synthesis and genomic rearrangement and paralleled by increased energy demand. These phenomena in familial as well as in sporadic cases of neurodegenerative disorders are linked to aberrant protein expression; in the latter, multifactorial frailty does not exclude that a high metabolic demand may exacerbate the toxic nature of various proteins. This could be the trigger of the molecular cascade leading to neurodegenerative disorders [2].

Glial cells (oligodendrocytes, astrocytes, and microglia) have been shown to play a paramount role in synapse development and functioning both in adaptive and maladaptive plasticity. An emerging factor that improves this model is the extracellular matrix (ECM), an intricate and highly organized scaffold of molecules once considered a simple structural element but rediscovered as a functional and plastic environment for intercellular communication [3,4]. However, scientists are nowadays so fascinated by the stereotyped neuronocentric view of brain disorders to relate the ECM component heparan sulfate proteoglycans (HSPGs) quite exclusively to the formation of neurofibrillary tangles [5]. The HSPGs role as a component of the ECM, thereby altering processes, such as cell adhesion, immune cell infiltration, and angiogenesis, has been neglected [5].

First, we introduce the CNS frailty coupled with energy demand, which could produce metabolic dysfunctions in both familial and sporadic forms of neurodegenerative diseases [6,7]. An integrated model of synaptic plasticity is then discussed that could encompass all cellular and extracellular elements and may be used to design experiments with a systems biology method. The neurovascular unit (NVU) and the extravascular matrix are discussed separately as the emerging issue of metabolic failure. The proper modeling of synaptic plasticity could pave the way for designing treatments acting on both the prevention of damage and the reparation processes of neurological diseases.

## 2. The Region-Specific Metabolic Coupling

A surprising but poorly understood feature of most neurodegenerative diseases is that they affect discrete neural systems in a somewhat coherent regional pattern, while other brain regions are totally spared or only affected in very advanced stages of the disease. In their study, Grothe et al. show that regional vulnerability in sporadic Alzheimer’s disease (SAD) is closely related to distinct molecular features of the involved brain regions. The different regional vulnerabilities for amyloid deposition and neurodegeneration were associated with widely different molecular profiles corresponding to distinct biochemical pathways of cellular and non-cellular functions [5]. Brain regions vulnerable to amyloid have been reported to be comparatively characterized by low levels of expression of mitochondrial respiration genes and to low rates of oxidative phosphorylation, a process known as “aerobic glycolysis” [8]. However, some brain regions, such as the primary visual cortex, although characterized by high neuronal activity and high energy consumption, are generally spared from amyloid deposition [9]. The functional and electrophysiological measurements of cerebral activity in these areas and the somatosensory cortex have shown peculiar characteristics both in health and diseases [10,11,12,13]. This could support that vulnerability to protein misbehavior could relate to different factors and not primarily to neuronal/synaptic activity [14]. In a system such as the CNS, however, these processes are usually non-cell-autonomous, and the microenvironment can be important in modifying the cellular vulnerability [15]. Remarkably, the same aberrant protein can form different pathological structures in certain cell types [16]. Moreover, in the CNS, neurodegeneration is not exclusive of neuronal cells; thus, other factors could underlie the pattern in which non-neuronal cells accumulate misfolded proteins, paralleling the stages of intraneuronal pathology [17]. Finally, aberrant proteins and aging could alter the genomic composition of CNS cells, as in SAD, where the age-related increase in genomic complementary DNAs (gencDNA) variants was shown as a novel pivotal risk factor [18]. The gencDNAs manifest as thousands of distinct genomic variants derived from a cellular gene, which can undergo multiple retro-insertions into post-mitotic neuronal genomes and appear capable of being actively transcribed and translated to produce variant bioactive products that are relevant to both normal and diseased states [18].

Neuroanatomical regions exhibit differential vulnerability with orderly recruitment of brain regions to the disease progression in a stereotyped manner. Within these regions, different neuronal and glial populations show a differential vulnerability to dysfunctions, leading to phenotypic diversity. In familial as in sporadic forms, vulnerable neurons face resistant neighbors [19]. Moreover, the aberrant protein usually does not show higher gene expression in vulnerable regions or cell types. In Parkinson’s disease, the analysis of the Genotype-Tissue Expression (GTeX) data shows a median level of α-synuclein expression relatively low in the substantia nigra and a high level in the cerebellum, generally spared from the disease [19,20]. Even more in multiple-system atrophy (MSA), the oligodendrocytes contain cytoplasmic inclusions of filamentous α-synuclein correlated with disease in discrete brain regions [21].

There is a tremendous metabolic burden on dopaminergic neurons in the midbrain caused by the anatomical architecture (each neuronal process forms enormous complex arbors), Ca^2+^ homeostasis, and dopamine toxic neutralization [22,23]. However, many mechanisms are shared between the substantia nigra and ventral tegmental area (VTA), posing the question of the selective nigral susceptibility in PD. The differential metabolic demand and a more efficient Ca^2+^ buffering of the VTA seem to be the major factors; however, anatomical connections and non-neuronal cells also could influence this regionality [19]. Moreover, astroglial cells accumulate α-synuclein in Lewy Body Diseases (LBD) confrontable with the stages of intraneuronal pathology [17]. It is still unclear whether the prion-like nature of the misfolded proteins or the transfer of α-synuclein (oligomers or deposits) between neurons and astrocytes is a required step or an epiphenomenon.

In α-synucleinopathies, the inclusion, formation, and spread require not only dopaminergic cell-autonomous factors but participation from other non-cell-autonomous factors generated from the glial cell types, the composition of the extracellular matrix (ECM), and the breakdown of the blood-brain barrier (BBB). The regional vulnerability of neurodegenerative diseases, such as SAD or Parkinson’s disease, could be determined, in part, by specific systemic properties (anatomical and functional) of the affected neural networks [5,24]. Furthermore, the BBB dysfunction has been defined as an early biomarker of cognitive decline and early stages of SAD [25,26]. It was shown that patients carrying apolipoprotein E4 (APOE4), an identified genetic risk factor for AD, have higher BBB permeability in the medial temporal lobe and hippocampus compared with non-carriers even when cognitively healthy [27]. The BBB failure was more severe in carriers with cognitive impairment but unrelated to β-amyloid or tau concentrations (both consolidated AD biomarkers). The NVU damage measured in vivo, considering pericytes and platelet-derived biomarkers, predicted the future cognitive status in APOE4 carriers even after controlling the analysis for differences in Aβ and tau levels [27,28].

Moreover, in CAG-triplet diseases, as Huntington’s disease (HD) and spinocerebellar ataxia (SCA), also the regional expression of enzymes catalyzing the post-translational modifications of aberrant proteins has to be taken in account, as they could play a role in the development of neurodegenerative disorders [29]. Considering HD, a study of gene-expression profiling identified two proteins whose expression most strongly correlated with regional vulnerability: the Wnt inhibitory factor-1 (WIF-1) and the protein phosphatase 1 regulatory subunit 7 (PPP1R7) [30]. These are pioneering studies that are based on the idea that only a differential concurrence of specific factors may lead to differential cellular and regional vulnerability.

The analysis of the synaptic model needs to provide a comprehensive and updated physio- and pathological synopsis of processes to propose future experiments with a network biology approach.

## 3. An Integrated Model of Synaptic Plasticity

Synapses are the anatomic and functional unit underlying the complexity of brain functioning, subtended by the expression of hundreds of proteins that provide electrochemical coupling, structural organization, vesicle turnover, and intracellular signaling. The brain consumes a disproportionate amount of the body’s energy [31], and most of the energy used in the brain is dedicated to synaptic transmission. Energy in the CNS is mainly provided by glucose and generated through mitochondrial oxidative phosphorylation [32]. Moreover, synaptic energy utilization, as an integral part of synaptic plasticity, seems to be highly dynamic—via, for example, the process of trafficking mitochondria to synapses and increase in the utilization of AMPA receptors, which may thus double the postsynaptic energy consumption to potentiated synapses [33,34]. Nonetheless, the brain, as clearly stated by Iadecola, “seems to have a fundamental design glitch: it consumes a large amount of energy but lacks a reservoir to store fuel” [35].

### 3.1. Cellular and Extracellular Interactions

**Figure 1:**
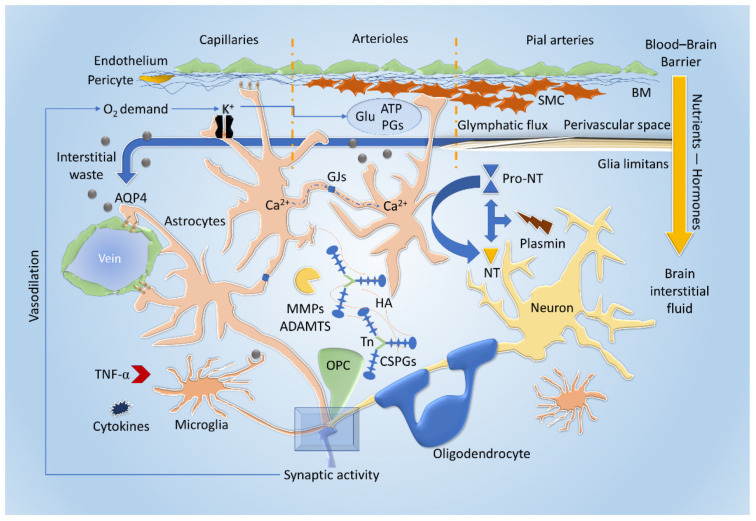
Metabolic coupling, extracellular signaling, and interstitial fluid dynamics. Synaptic activity regulates the release of K^+^ and augments O_2_ demand. These phenomena act directly on capillaries (endothelium and pericytes) and probably on astrocytes, increasing the blood flow particularly producing hyperpolarization to smooth muscle cells (SMC). These cells, relaxing, prompt vasodilation, which is also stimulated by direct gliotransmission that releases glutamate (Glu), ATP, and prostaglandins (PGs) with a Ca^2+^-mediated mechanism. The neurovascular coupling may also increase nutrient availability and hormones transport through the blood-brain barrier (BBB). The perivascular space from pial arterioles and tight-junction capillaries and their basal membrane (BM) are involved in convective fluxes (glymphatic) pathways that rely on astrocytic aquaporin-4 (AQP4) on astrocytic end-feet to clear interstitial wastes from the central nervous system (CNS) towards the perivascular venous side and subsequently dural lymphatics (not shown). Modifiers, such as matrix metalloproteinases (MMPs) and a disintegrin and metalloproteinase with thrombospondin motifs (ADAMTS), are produced by the different cellular elements, as for the chondroitin sulfate proteoglycans (CSPGs), tenascin (Tn), and hyaluronic acid (HA). The neurotrophins (NT) levels can be modified by the MMPs, by the neuronal plasmin, and astrocytic re-uptake, cleaving their pro-form (pro-NT) or degrading the mature protein. Microglial cells also contribute to NT secretion and tumor necrosis factor α (TNFα) and cytokines production to fulfill their role of innate immunity element while scanning the environment for molecular wastes. Oligodendrocytes and their precursors (OPC) are not only indispensable for myelination but are associated with learning and also contribute to the maintenance of the ECM.

**Figure 2:**
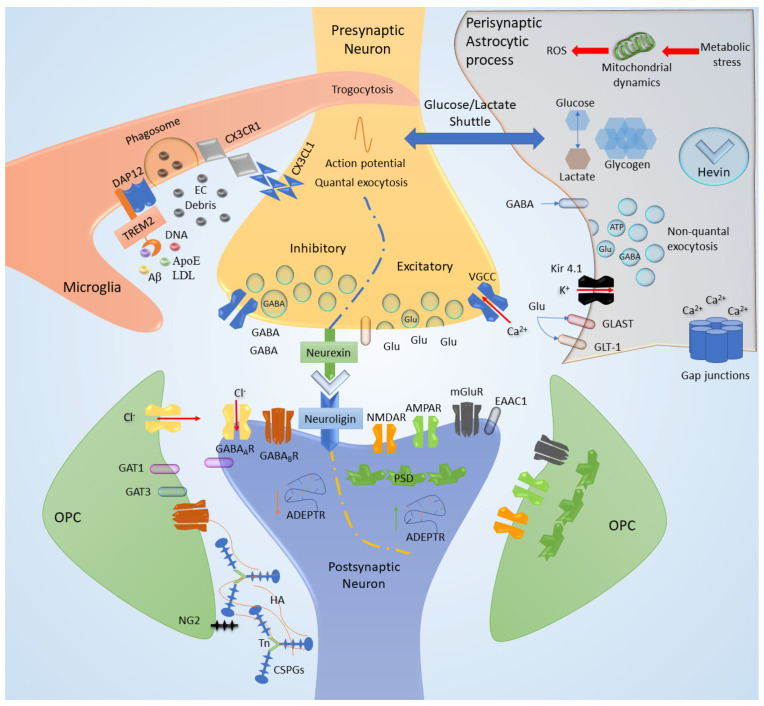
Synaptic cleft components. The schematic representation of a synapse with both excitatory and inhibitory assets emphasizes the contribution of cell adhesion between synaptic terminals (neurexin/neuroligin complex), the stabilization of astrocytic hevin, and the extracellular (EC) matrix proteins functional anchorage. The role of ionic fluxes and ionotropic/metabotropic receptors is peculiarly regulated considering each depicted element. These transmitters can be secreted through quantal vesicular exocytosis through neuronal activity and via non-quantal secretion considering the so-called “gliotransmission”. Moreover, astrocytes are responsible for metabolic coupling and major mitochondrial dynamics. The microglial scavenger and immunologic role through specific receptors (the sensome) is widely more complex than the showed exemplification. Here, we depicted the triggering receptor expressed on myeloid cells 2 (TREM-2) and the DNAX-activation protein of 12KDa (DAP12), which can interact with proteins, lipoproteins and DNA. The fractalkine receptor/ligand (CX3CR1/CX3CL1) aids the association between the neuron and microglia at the synaptic level, where the partial engulfment of spines or axons is called trogocytosis. Eventually, the synaptic scaffolds both, considering excitatory postsynaptic densities (PSD) or inhibitory arrangement of the cytoskeleton, can be regulated by non-protein elements, such as the activity-dependent transported long non-coding RNA (ADEPTR). GAT, GABA transporter; mGluR, metabotropic glutamate receptor; Kir, inward rectifying potassium channels; NG2, neural/glial antigen 2; EAAC1, excitatory amino acid carrier 1; GLAST, glutamate/aspartate transporter; GLT-1, glutamate transporter 1; HA, hyaluronic acid; Tn, tenascin; CSPG, chondroitin sulfate proteoglycan; AMPAR, AMPA receptor; NMDAR, NMDA receptor; VGCC, voltage-gated calcium channels.

The reliance of the brain on energy is reflected well in neurodegenerative disorders of aging (NDAs), in which energy-utilization pathways are progressively disrupted on multiple levels (e.g., glucose uptake, mitochondrial functioning, and axonal transport) [32]. In states of intense neural activity with particularly high energy demands, such as LTP (Long Term Potentiation), L-lactate seems to act as a key substrate for energy production. This process is mediated through astrocytes—the Astrocyte Neuron Lactate Shuttle (ANLS) neuro-energetic model proposes that synaptic activity triggers the production of L-lactate through aerobic glycolysis in astrocytes [36]. Glycogen, from which metabolic processes produce L-lactate, is stored in astrocytes, thus expressing their role as energy reservoirs [37]. Glycogen granules in astrocytic processes seem to be distributed preferentially around synapses, suggesting the potential role of lactate as both an energy substrate and as a signaling molecule for plasticity (Figure 1 and Figure 2) [37]. These features allow the CNS to reshape itself through the pruning of new synapses or by maintaining stable circuits while eliminating redundancies or unused connections based on an experience-dependent paradigm [38,39]. Synaptic plasticity is comprised of different mechanisms occurring in concert as a result of multiple levels of influence [40]. A key actor in synaptic plasticity, which exemplifies the different mechanisms and factors involved, is the dendritic spine. Dendritic spines remain highly dynamic in mature neurons and can undergo changes in number, size, structure, and composition, all of which contribute to the formation and experience-dependent optimization of neuronal circuits [41,42]. Spine plasticity occurs through developmental mechanisms during formation and through activity-dependent plasticity mechanisms, which occur through different levels of influence. Hebbian synaptic plasticity occurs on the synaptic level, through synaptic activity and frequency of stimulation [43], including LTP and long-term depression (LTD). Homeostatic plasticity occurs on the circuit level and refers to the self-regulation of neurons in response to circuit activity—a form of which is termed synaptic scaling, in which neurons reduce the strength of their synaptic connections in response to global elevated circuit activity [44]. On the cellular level, these influences are realized through various mechanisms. A key mechanism is the changes in surface AMPA receptor (AMPAR) density [45]. AMPARs and NMDARs are glutamate receptors, the main mediators of excitatory neurotransmission in the brain, and play a central role in the structure and function of spines; they influence spine number, size, motility, and synaptic stability number and strength [40]. The elements of synaptic plasticity exemplified through spine plasticity involve and are dependent on numerous intracellular and extracellular pathways and actors, many of which have been identified in numerous studies, which investigated their function in synaptic plasticity as well as their role in neuropsychiatric diseases. Cell adhesion molecules (CAMs), specifically Neurexins, scaffold proteins in spines, of which SHANK-3 and ANK3 are prominent examples, voltage-gated calcium channels (VGCCs), and their subunits and regulatory signaling proteins, including small GTPases and the proteins with which they interact (e.g., RAS), influence AMPAR membrane insertion, metabolic glutamate receptors (mGLUR) expression, spines size, and density [40] and more recently, the intronic long noncoding RNA (lncRNA)—termed ADEPTR—mediates structural plasticity at the synapse (Figure 2) [46].

Research has been oriented for decades to the dissection of single-element contribution (cellular or extracellular) to synaptic plasticity. The single-cell contribution, however, is difficult to prove and represents the limitations of a comprehensive synaptic model. The evidence of the past decades and the limits of the single-cell-based model could be demonstrated considering the role of astrocytes in established synaptic plasticity. The perturbations of astrocytes reveal their systemic interactions with neuronal and other glial cells, modifying the stability of the NVU and determining changes of the ECM composition and energetic supply (Figure 1 and Figure 2).

As an integral part of the NVU, astrocytes have an important role both in blood flow regulation and as part of the BBB (i.e., regulating substance entry into the brain interstitial fluid or BISF) [35,47]. The linking of neural activity to microvascular function, enabling coordination of blood flow and substance transfer across the BBB and into the BISF in response to neural stimulation, is termed “neurovascular coupling” (Figure 1). Astrocytes have been proposed to play a key role in this function. Several mechanisms through which astrocytes may influence the microvasculature were investigated and proposed [48,49,50]. Some studies suggested that Ca^2+^ increases, triggered by neural activity via glutamate, could influence the diameter of adjacent microvessels through various upstream and downstream mechanisms, involving astrocytic secretion of the so-called “gliotransmitters”, which may include glutamate, COX products (prostaglandins), ATP, and other products, thus inducing vasoconstriction or vasodilation and influencing blood flow (Figure 1) [51,52,53]. Moreover, the release of K^+^ and the increased O_2_ request during synaptic activity can directly act on endothelium and pericytes at capillary level or through the hyperpolarization of arteriolar smooth muscle cells (SMC). [35] Apart from being a mechanism to increase oxygen and nutrient availability, neurovascular coupling may also regulate movement of hormones and peptides across the BBB [54]. Astrocytic end-feet, the final barrier for regulating the passage of substances from the blood to the BISF [55], may play an important role in this function.

Insulin appears to reach the brain primarily through the BBB [56,57], and studies have demonstrated that glutamate stimulation of astrocytes increases insulin transcytosis across the BBB, increasing insulin passage in a mechanism independent of blood flow (Figure 1) [47].

Astrocytes are fundamental for synaptic stability, expressing hevin (high endothelial venule protein), which reinforces the bonding of the pre-and postsynaptic neurexin/neuroligin complex (Figure 2) [39]. Moreover, astrocytes could influence neuronal firing by (i) reducing potassium conductance, through inward rectifying potassium channels (Kir), especially Kir 4.1; (ii) the reuptake of neurotransmitters (glutamate and GABA) through the expression of specific transporters, particularly glutamate transporter 1 (GLT1) and glutamate/aspartate transporter (GLAST) or GABA transporters (GAT), particularly GAT1 and GAT3, the latter being able to regulate the astrocytic intracellular concentration of calcium; and (iii) the ability to secrete, in a calcium-dependent fashion, glutamate, GABA, D-serine, and purinergic mediators through a gliotransmission, which differs from the quantal release of neurotransmitters [4] (Figure 2).

Aquaporin-4 (AQP4) channels, expressed on the astrocytic membrane, are involved in osmotic regulation, which seems to be necessary for the removal of ECM waste through an active filtration process conveying debris to the NVU and cerebrospinal fluid (CSF) through the glymphatic system, and could partly subtend the wakefulness/sleep cycles and neurodegeneration (Figure 1) [58].

Moreover, astrocytes regulate the concentration of neurotrophins (NTs), such as the nerve growth factor (NGF) and the brain-derived neurotrophic factor (BDNF), in the ECM by both secreting and actively modifying their pro-forms [38]. NGF is a key molecule in differentiation and maintenance of specific neuronal populations [59,60] through various processes, such as autophagy [61], changes in intracellular Ca^2+^ concentrations [62,63], and changes in energetic profiles; specifically, NGF determines several effects on mitochondria biogenesis, modeling, distribution, and functioning [15]. A single human astrocyte could cover several synapses, and clusters of these cells are interconnected with gap junctions through connexins (Cx), particularly Cx30 and Cx43, forming astrocytic domains that could synchronize or reciprocally inhibit specific neuronal circuits without a strict proximity rule through Ca^2+^ waves. The Cx seem to be crucial, together with neurovascular coupling, for metabolic supply (Figure 1) [4]. The high structural motility (functional reshaping in a timescale of minutes) of the astrocytic cytoskeleton and its high sensitivity to plasticity processes with differential expression, among others, of the glial fibrillary acidic protein (GFAP) make these cells capable of releasing gliotransmitters for even up to one hour after neuronal firing while storing information over time to reinforce synaptic circuitries [4]. Despite their importance, the extent of astrocytic enwrapped synapses is quite variable, ranging from an estimated 15% to 90%, depending on the considered brain area as well as functional states. These data support the necessity of a multi-factorial model with an important role defined for astrocytes that could be validated by analyzing the other components of the system [4]. Moreover, the astrocytic enwrapping of the synapse could be pivotal in determining the spatial diversity of neurodegenerative processes and the temporal connection between metabolic functions and synaptic plasticity, these cells being involved in all the aforementioned systemic functions. As a consequence, regional energy failure and aberrant proteins deposition could occur.

Microglia, which have multiple functions in the CNS, have been shown to play a role in synaptic plasticity, assisting in synapse pruning, relocation, and elimination with a ubiquitous distribution throughout the CNS [64]. The scavenger role of microglia led to a simplistic parallelism with the resident macrophagic system of other tissue. However, microglia do not differentiate from circulating monocytes and do not share the same embryonic origin [65]. Microglial cells function as highly dynamic sensors that can check the whole CNS tissue in a timeframe of hours, a continuous process that exceeds beyond a scavenger function. Through high-density receptors (called “sensome”), microglia can scan the ECM and NVU homeostasis, synaptic activity, and the functional state of other cells. Among these receptors, there are the complement receptor CR3 interacting with the immune system and the NVU, the TREM-2 receptor expressed on myeloid cells 2, and the DNAX-activation protein of 12 KDa (DAP12) that activates phagocytosis [66] (Figure 2).

The interaction with neurons is mainly mediated by purinergic receptors and CX3CR1 for neuronal fractalkine (CX3CL1). Through CX3CR1 and the complement components C1q and C3, microglial processes interact with neurites and synapses, although the precise mechanisms have yet to be elucidated [66]. A peculiar mechanism demonstrated in microglia is the trogocytosis by which their processes enwrap portions of axons to guide their growth and eliminate presynaptic connections (Figure 2) [67]. Signaling pathways are activated by secretion of molecules like NTs, particularly the BDNF, cytokines, such as the tumor necrosis factor α (TNFα), components of the ECM and micro ribonucleic acids (microRNAs) that could be released by the typical exocytosis (vesicle-membrane anchoring), or through the extracellular vesicles (EV) formation. BDNF is involved in neurotransmission modulation (GABA excitatory/inhibitory conversion), transmitter reuptake, and reactive glial activation [4] (Figure 1).

Microglial cells are involved in synaptic plasticity, waste elimination, and inflammatory reaction, being good candidates to prompt or sustain regional degenerative processes. These cells seem to be less involved in active energy supply; nonetheless, the rupture of the NVU homeostasis mediated by maladaptive microglia activation could start the functional starvation of the system.

Oligodendrocytes and their precursor cells (OPCs) are key players in myelination and white matter physiology and pathology [68]. OPCs are known in the literature to express the neural/glial antigen 2 (NG2). The platelet-derived growth factor (PDGF, secreted from both neurons and astrocytes) seems to be the main inducer of OPC proliferation acting in association with ECM and cellular integrins (i.e., the phosphorylated form of αVβ3, Tenascin-C (TnC) and NG2) (Figure 2). Other than the role in myelin homeostasis, OPCs express a variety of neurotransmitter receptors and voltage-gated channels and are the only known glial cells that synapse with neurons both with glutamatergic and GABAergic cells (Figure 2). OPCs maintain high motility, possibly providing neurotrophic factors, regulating axonal outgrowth, and monitoring neuronal excitability and axonal firing. The induction of activity-dependent oligodendrocyte maturation seems to be fundamental for learning, as elegantly demonstrated for motor tasks [69]. In particular, OPCs also express excitatory postsynaptic densities (PSD) and showed that the same receptors could have emerging properties. For instance, the GABA type A receptor (GABAAR) causes Cl^−^ efflux and depolarization in OPC, whilst the opposite happens in neurons (Figure 2).

Oligodendrocytes have been shown to have the potential role of builders of the extracellular environment and to be paramount in synaptic plasticity. As aforementioned, glial cytoplasmic inclusions (GCIs) composed of filamentous alpha-synuclein are recommended as the defining morphological feature of MSA [70]. A systems biology model could improve knowledge of oligodendrocytes, furthering the understanding of acute or chronic diseases that do not involve the formation or maintenance of white matter.

These data strengthen the notion of the interplay between different cellular and non-cellular components supported by the penta-partite model. This is pivotal in the systems biology approach, focusing on the neuronal/glial/vascular networks, which may as well be inadequate, as shown, for instance, by promising yet partial results obtained reducing reactive gliosis [38].

### 3.2. The Emergent Roles of the Extracellular Matrix and the Neurovascular Unit

Synaptic plasticity processes are subtended by both structural and functional changes. The ECM and NVU are responsible for structural changes and support the availability of signaling molecules, nutrients, and growth factors essential for the precise temporal and spatial progression of functional interactions. All cellular components are involved in the formation of these bio-scaffolds in both health and diseases.

One example of ECM relevance is the role of the Thrombospondins 1 and 2 (TSP-1/2) in synaptogenesis and axonal sprouting, especially in the context of cortical plasticity after stroke [71]. The two molecules are extracellular glycoproteins, stabilizing the ECM by interacting with other factors, such as laminin and fibronectin. In their paper, Liauw et al. [72] showed that TSP 1 and 2 are needed for synaptic plasticity after stroke.

ECM components, such as hyaluronan and Chondroitin Sulfate Proteoglycans (CSPGs), also play a role in neural plasticity and axon regeneration by physically regulating these processes (Figure 2) [73]. Hyaluronan, along with other ECM parts, stabilizes dendritic spines, thus affecting dendritic spine dynamics and may also affect synaptic plasticity by binding to components of the dendritic spine [73].

Experiments conducted to determine the contribution of single-cell elements in ECM composition have failed to accomplish significant results [74]. Matrix metalloproteinases (MMPs) and a disintegrin and metalloproteinase with thrombospondin motifs (ADAMTS) are proteases secreted by both neurons and glia to reshape the proteoglycan/glycoprotein and hyaluronan structure of the ECM in response to perturbation of homeostasis or as physiological turnover (Figure 1) [3].

Perineuronal nets (PNNs) are a specialized form of ECM, surrounding certain neuronal bodies that allow a selectively permeable filter to modulate the formation of novel connections and retain signaling molecules, such as semaphorin/plexin system, probably to prevent circuitry miswiring or redundancies. Chondroitin sulfate proteoglycan 1 (CSPG1), also known as aggrecan, seems to be one of the major PNN components found in the proximity of developing synapses. The increased expression of CSPGs, TnC, TnR, and hyaluronic acid may inhibit axon elongation during reactive gliosis. Differential deposition of CSPGs has been observed in CNS diseases, for instance, the overexpression of neurocan and phosphacan paralleled by the decrease of brevican and neurocan following ischemic damage.

The production of NTs can occur following the activation of the secreted pro-NTs in the ECM by serine proteases, like plasmin and MMPs (e.g., MMP-7 and MMP-9) (Figure 1) [75].

Alteration of endogenous NGF metabolism, characterized by decreased levels of mature NGF, increased proNGF, and increased activity of NGF-degrading MMPs, correlate to microglial astrocytic responses indicative of reactive gliosis [76]. Plasmin, exclusively expressed by neurons in the CNS, is normally inactive as plasminogen and can be activated through proteolytic cleavage mediated by tissue plasminogen activator (tPA).

The linkage between ECM and cellular components is mediated by CAMs. The astrocytic/neuronal crosstalk is mainly mediated by synaptic CAM (SynCAM), hevin/neurexin/neuroligin complex (Figure 2), and a member of the leucine-rich repeat transmembrane proteins (LRRTM). LRRTM proteins bind neurexins, induce presynaptic differentiation, and help regulate receptor composition. Neuronal CAM (NCAM), on the other hand, is critical for visual cortex development: visual stimuli induce its post-transcriptional modification (i.e., polysialylation) to enhance the homophilic interactions across the synapse. Polysialylation of SynCAM in certain brain areas is associated with OPCs and synapse maturation [4].

Structurally, the NVU is comprised of vascular elements (brain endothelial cells, basement membrane, pericytes, and smooth muscle cells), ECM, microglia, astrocytes, and neurons (Figure 1). The NVU in its complexity allows CNS metabolic supply, waste disposal, and immunological access and is regulated by the bidirectional exchange between the systemic circulation and the brain parenchyma through the BBB (constituted of tight-junction endothelium, the basement membrane, pericytes, and astrocytic end-feet) [75]. Pericytes, in particular, seem to be important for BBB permeability to water and solutes, regulating endothelial transcytosis, possibly altering gene expression of endothelial cells, and inducing the membrane polarization of astrocytic end-feet around vessels [77].

The microglia, on the other hand, are the cells of the innate immune response and therefore the core of the immune system. They preserve the immune stability of the CNS by interacting with neuroimmune regulators (NIRegs), such as CXCL1, CD47, and CD200, presented by resident cells and by inhibiting the complement activation through factor H, CD46, and CD59 in the lack of endogenous perturbations [75].

Moreover, cytokine signaling is physiologically silenced by the constitutive expression of the suppressor of cytokine signaling (SOCS). Neuro-immune response and the breakdown of NVU integrity could be induced by the activation of the proteinase-activated receptors (PARs) by thrombin. For this reason, thrombomodulin and serpins are considered also NIRegs. In particular, PAR1 can be activated by thrombin, MMP1, plasmin, and activated factor X (FXa) by the canonical cleavage and tethered ligand exposure, leading to neurotoxic sequelae through the rat sarcoma protein (Ras)-related protein A (RhoA) pathway. Low concentrations of thrombin, complexed with activated protein C (aPC) and its endothelial receptor (EPCR), on the other hand, exposes an alternative site of cleavage, the so-called biased agonism, through the Ras-related C3 botulinum toxin substrate 1 (Rac-1) pathway, which seems to be neuroprotective. As an untethered ligand of PAR1 and PAR4, C4a seems to activate intracellular phospholipase C (PLC) and calcium release, supporting the role of the NVU and the neuro-immune system in synaptic plasticity [75]. NVU and CSF dynamics are important factors in the directional flow from the arterial perivascular space to venous drainage through brain ECM, a process involved in CNS waste clearing, especially during the sleep, possibly through a cyclic increase of the interstitial medium and reduced noradrenergic tone by the locus coeruleus [58].

The activation of signaling pathways from the ECM and NVU that encompass these cellular elements are majorly conveyed by integrins. The integrin family is the most studied among cellular receptors of glycoproteins of the ECM, and the key element of interaction seems to be TnC. The integrins α8β1, α9β1, αvβ6, and αvβ3, recognizing the fibronectin type III repeats, can be paramount contributors to maintenance and differentiation of neuronal cells also cooperating with other signaling mechanisms, such as the Notch pathway [78].

TnC can act as a bridge in this system (Figure 1); it is a hexameric glycoprotein, interacting with CSPGs (neurocan, aggrecan), HSPGs (syndecan), and with both receptors (e.g., epidermal growth factor receptor) and growth factors, such as transforming growth factor β (TFG-β), PDGF, neurotrophin-3 (NT-3), wnt3a, and fibroblast growth factor 2 (FGF-2).

The activated pathways are majorly involved in adhesion, migration, differentiation, self-renewal, and proliferation through the activation of focal adhesion kinase (FAK), mitogen-activated protein kinase MAPK, and protein kinase B (PKB) or PKC [78].

The importance of TnC/integrin interaction has been proved also in regenerating properties, as following spinal cord or nerve injury (dorsal rhizotomy, dorsal column crush) with the reintroduction of α9 integrin, normally downregulated in adult CNS, aiding neurite outgrowth and sensory regeneration [79].

The expression of integrins by non-resident immune cells, such as the αLβ2, also known as Lymphocyte function-associated antigen 1 (LFA-1) or CD11a/CD18, can account for the pathogenic effects of NVU disruption, as shown in the early neutrophil extravasation in an AD model. LFA-1 inhibition, neutrophil depletion, and LFA-1 knockdown reduced both the pathological findings and the neurocognitive dysfunctions [80].

## 4. The Burdensome Variable Energy Key Issue

Energy demand required by brain activity is highly dynamic and diverse, creating the need, in turn, for highly modulated blood supply to the right time and place and in the right amount. In this context, the concept of the NVU emerged, challenging the once-held conceptual dichotomy between neuronal and blood vessel functioning, instead emphasizing their symbiotic relationship, and their developmental, structural, and functional interdependence [35].

The most surprising data reported in recent years to support the definition: “the key problem of heavy variable energy” is that the region-specific neuronal vulnerability is a response of astrocytic metabolism. Astrocytes in each brain region, especially under altered energy demand/resource conditions, adapt by metabolically reprogramming their mitochondria to use endogenous non-glycolytic metabolites as an alternative fuel. In the striatum, a region enriched in fatty acids (FA), in case of an energy shortage, the astrocytes reprogram mitochondrial metabolism, utilizing FA as an energy source but increasing tremendously the reactive oxygen species (ROS) (Figure 2). Oxidative stress has been investigated in CNS diseases as a major contributing factor [81,82,83]. In the cerebellum, in similar conditions, the astrocytes utilize amino acids (AA), abundant in this region. The AA are employed as precursors for glucose generation through the pentose phosphate shunt or gluconeogenesis pathways [84].

Remodeling of entire brain neuronal networks has been reported as a consequence of a specific regional failure in the anterior forebrain. The mitochondrial respiratory chain measured as cytochrome oxidase activity revealed by cross-correlations among different brain areas an altered cross-talk in different brain regions [85].

Astrocytes, by virtue of their paramount role in brain tissue homeostasis, are highly reactive in inflammatory states [86,87,88] and play a crucial role in the adaptive processes following inflammatory insult to brain tissue. Processes induced by inflammatory signaling in the astrocyte are the mitochondrial dynamics, of which mitophagy, a specific form of macroautophagy, is an important element [89,90,91]. Mitophagy enables the regulation of mitochondrial turnover, possibly segregating damaged mitochondria from the healthy network [92]. Inflammatory stimuli induce mitochondrial network fragmentation and impaired respiration rate, a response mediated by the pro-fission protein Drp1, which requires induction of autophagy for its resolution [91]. It is suggested that a timely activation of autophagy is critical to safeguard mitochondrial function in astrocytes during a proinflammatory response. Furthermore, it seems that mitochondrial dynamics are heterogeneous depending on the type and severity of the insult and differ, for instance, between lesion core and penumbra in ischemic states [91].

Very recently, it has been reported that the tumor microenvironment (TME) can be both favorable to the metabolism of cancer cells but also harmful to the metabolism of lymphocytes. This suggests that metabolic adaptations determine whether a cell thrives or is hampered by TME. TME has been shown to directly paralyze the glucose metabolism of NK cells via lipid-peroxidation-associated oxidative stress as a central mechanism to inhibition. Therefore, metabolic flexibility appears to be a key determinant of NK cell fate in TME as well. NK cells showing complete substrate flexibility are not only metabolically active but paradoxically increase their tumor-killing in response to hostile TME and nutrient deprivation [93]. These processes play a key role also in the behavior of the glioblastoma (GBM), one of the most aggressive tumors to treat [94].

## 5. Future Perspectives

**Figure 3:**
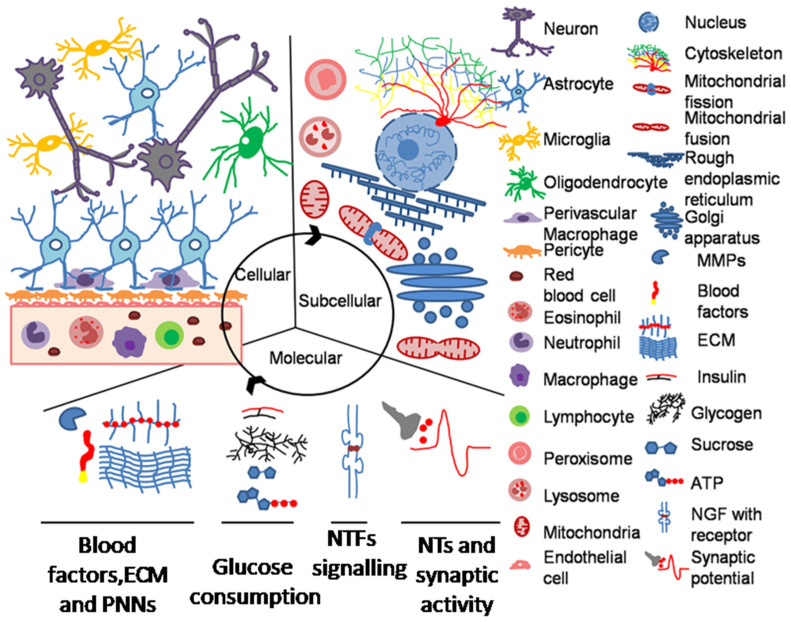
Cellular, subcellular, and main molecular components to be considered in synaptic plasticity modeling on a spatiotemporal concurrency. ECM, extracellular matrix; PNN, perineuronal nets; NTFs, neurotrophins; NTs, neurotransmitters.

Understanding the regionally heterogeneous distribution of pathological changes in neurodegenerative diseases and the shared characteristics of those neural systems that are more vulnerable in specific pathologies than others is fundamental for understanding the pathogenesis of each disease. The presented data suggest that a paradigm shift in synaptic plasticity modeling is necessary. The understanding of systemic complexity and the design of novel experiments should consider the functional and structural relations between cellular, subcellular, and molecular components participating in the plasticity phenomena (Figure 3). Neurons alone, it seems, are unable to efficiently develop, transform, or strengthen synaptic circuits without interactions with glia, ECM, and NVU.

We reviewed the main pathways that we propose should be studied to develop a comprehensive knowledge of synaptic plasticity.

The recent gathering of the so-called omics data (e.g., genomic, transcriptomic, proteomic, epigenomic, etc.) brings to attention complex scenarios of various physiological and pathological perturbations that need a reiterative and modular approach to be interpreted. The integration of these modules into a dynamic and integrative model, such as the penta-partite synapse, may allow the reading of big-data flows from molecular networks to subcellular, cellular, and extracellular distributions. The theoretical model of the synapse and its key elements should be tested through computational predictions to identify possible network perturbations to be further verified with biological experiments.

Hence, a modular systems biology approach could permit the structuring of these processes and may allow understanding the regulatory rationality belonging to different but interconnected domains, leading to the identification of new targets for both prevention and treatment of neurological diseases.

## Data Availability

Not applicable.
